# Cognitive therapy and eye movement desensitization and reprocessing for reducing psychopathology among disaster-bereaved individuals: study protocol for a randomized controlled trial

**DOI:** 10.1080/20008198.2017.1388710

**Published:** 2017-10-18

**Authors:** Lonneke I. M. Lenferink, Eline Piersma, Jos de Keijser, Geert E. Smid, Paul A. Boelen

**Affiliations:** ^a^ Department of Clinical Psychology and Experimental Psychopathology, Faculty of Behavioral and Social Sciences, University of Groningen, Groningen, The Netherlands; ^b^ Department of Clinical Psychology, Faculty of Social Sciences, Utrecht University, Utrecht, The Netherlands; ^c^ Foundation Centrum '45, Diemen, The Netherlands; ^d^ Arq Psychotrauma Expert Group, Diemen, The Netherlands

**Keywords:** Grief, bereavement, disaster, stress, psychopathology, treatment, aflicción, duelo, desastre, estrés, psicopatología, tratamiento, 哀痛, 丧亲, 灾难, 压力, 精神病理学, 治疗, • Confrontation with a traumatic loss is a risk factor for the development of psychopathology.• Research into the effectiveness of grief treatment is scarce.• The effectiveness of cognitive therapy with EMDR versus a waiting list control group in reducing psychopathology levels in traumatically bereaved people will be examined.• Possible mechanisms of change during treatment will be studied.

## Abstract

**Background**: Confrontation with a traumatic (e.g. disaster-related) loss is a risk factor for the development of psychopathology, including symptoms of prolonged grief (PG), posttraumatic stress (PTS), and depression. Although interventions have been developed for reducing post-loss psychopathology, more research into the effectiveness of treatment is needed to improve care for bereaved persons. Cognitive therapy (CT) and eye movement desensitization and reprocessing (EMDR) have been shown to be effective in trauma-exposed populations. We hypothesize that CT and EMDR are also effective in reducing symptoms among people exposed to traumatic loss.

**Objective**: In this article we describe the rationale of a randomized controlled trial (RCT) to examine (1) treatment effects of CT and EMDR for reducing PG, PTS, and depression among traumatically bereaved people, and (2) the associations between improvements in PG, PTS, and depression symptoms on the one hand and tentative mechanisms of change, including a sense of unrealness, negative cognitions, avoidance behaviour, and intrusive memories, on the other hand.

**Method**: A two-armed (intervention versus waiting list controls) RCT will be conducted. Participants will be asked to fill in questionnaires prior to treatment, during treatment, and one, 12, and 24 weeks post-treatment. Potential participants are people who have lost one or multiple significant other(s) in the Ukrainian plane disaster in 2014 with clinically significant levels of self-rated PG, PTS, and/or depression. Multiple regression, including analysis of covariance, and multilevel regression analyses will be used.

**Discussion**: There is a need for treatment for psychopathology following traumatic loss. Strengths of this study are the development of a treatment that targets grief and trauma-related complaints and the examination of potential mechanisms of change in CT and EMDR. Bereaved people, clinicians, and researchers could benefit from the results of this study.

## Abstract (es)

**Planteamiento**: Confrontar una pérdida traumática (p.ej., una pérdida relacionada con un desastre) es un factor de riesgo para el desarrollo de una psicopatología, tal como síntomas de duelo prolongado (DP), estrés postraumático (EPT) y depresión. Aunque se han desarrollado intervenciones para reducir la psicopatología post-pérdida, se necesita más investigación sobre la eficacia del tratamiento para mejorar la atención a las personas en duelo. Se ha demostrado que la terapia cognitiva (TC) y la desensibilización y reprocesamiento por movimientos oculares (EMDR) son efectivas en poblaciones expuestas a trauma. La hipótesis plantea que la TC y EMDR también son eficaces en la reducción de síntomas en personas expuestas a una pérdida traumática.

**Objetivo**: En este artículo se describe la justificación de usar de un ensayo controlado aleatorio (ECA) para examinar: a) los efectos del tratamiento de la TC y EMDR para reducir el DP, el EPT y la depresión en personas con un duelo traumático y b) las asociaciones entre las mejorías del DP y los síntomas de depresión, por un lado, y los mecanismos tentativos de cambio, que incluyen una sensación de irrealidad, cogniciones negativas, comportamiento de evitación y recuerdos intrusivos, por otro lado.

**Método**: Se llevará a cabo un ECA de dos brazos (intervención frente a lista de espera). Se les pedirá a los participantes que rellenen cuestionarios antes del tratamiento, durante el tratamiento, y una, 12 y 24 semanas después del tratamiento. Los participantes potenciales son personas que han perdido uno o varios seres queridos en el desastre aéreo ucraniano en 2014 con niveles clínicamente significativos de auto-puntuaciones de DP, EPT y/o depresión. Se usará una regresión múltiple, que incluye un análisis de covarianza, y un análisis de regresión multinivel.

**Discusión**: Existe necesidad de tratamiento para la psicopatología después de una pérdida traumática. Los puntos fuertes de este estudio son el desarrollo de un tratamiento que se centra en el duelo y las quejas relacionadas con el trauma, y el examen de los posibles mecanismos de cambio en TC y EMDR. Las personas en duelo, los clínicos y los investigadores podrían beneficiarse de los resultados de este estudio.

## Abstract (zh)

**背景**：遭遇创伤性丧失（如灾难相关的丧失）是精神病理发展的风险因素，包括延长哀伤（PG）症状、创伤后应激（PTS）和抑郁。尽管已经有减少丧失后精神病理的干预方法，还需要更多关于治疗效果的研究来改善对丧亲者的关怀。已经发现认知疗法（CT）和眼动脱敏与再加工（EMDR）对创伤暴露人群的效果。我们假设CT和EMDR在减少暴露于创伤性丧失者症状上也同样有效。

**目标**：在本文中我们描述了一个随机对照试验（RCT）的原理用来探究：a）CT和EMDR在创伤性丧亲群体中减轻PG、PTS和抑郁的治疗效果；b）PG、PTS和抑郁症状改进和潜在改变机制（包括非真实感、负性认知、回避行为和闯入记忆）的关联。

**方法**：进行一个双组设计（干预vs等待控制组）RCT。被试会被要求在治疗前、治疗中、治疗后的1、12、24周填写问卷。潜在被试在2014年乌克兰空难中失去1个或者多个重要他人，并在自评PG、PTS和/或抑郁上达到临床显著水平。使用多重回归包括协方差分析，和多水平回归分析进行统计。

**讨论**：在创伤丧失后需要进行心理病理治疗。本研究的优势在于发展一种针对哀痛和创伤相关的主诉的治疗方法，并探究CT和EMDR变化的潜在机制。丧亲者、临床工作者和研究人员都可以从本研究结果中获益。

Acute grief reactions, including separation distress (e.g. yearning for the deceased) and cognitive (e.g. confusion about one’s role in life), emotional (e.g. feeling stunned), and behavioural (e.g. avoidance of reminders of the loss) symptoms are common responses to the loss of a loved one. When these complaints persist or increase and are associated with significant distress and impairments in daily functioning, a diagnosis of Prolonged Grief Disorder (PGD; Prigerson et al., 2009) may apply. PGD will likely be included in the 11th edition of the International Classification of Diseases (ICD-11; Maercker et al., 2013). Also, a diagnosis of persistent complex bereavement disorder (PCBD) may be considered in individuals with persistent and debilitating grief-related distress; PCBD has been included as condition for further study in the fifth edition of the Diagnostic and Statistical Manual for Mental Disorders (DSM-5; APA, 2013). PGD and PCBD capture the same diagnostic entity (Maciejewski, Maercker, Boelen, & Prigerson, 2016). Based on a meta-analysis, one out of 10 persons confronted with the non-violent loss (e.g. illness) of a significant other is at risk for developing PGD (Lundorff, Holmgren, Zachariae, Farver-Vestergaard, & O’Connor, 2017). Although symptoms of prolonged grief (PG) are often associated with elevated levels of posttraumatic stress (PTS) and depression, factor-analytic studies showed that these phenomena are distinguishable (Boelen, van de Schoot, van den Hout, de Keijser, & van den Bout, 2010; O’Connor, Lasgaard, Shevlin, & Guldin, 2010; Prigerson, Bierhals, Kasl, & Reynolds, 1996).

Being confronted with a traumatic loss (e.g. homicide/accident/disaster-related loss) has been associated with higher PG, PTS, and depression levels compared with non-violent loss (Boelen, de Keijser, & Smid, 2015; Kaltman & Bonanno, 2003; Kloep, Lancaster, & Rodriguez, 2014). Empirical work, drawing from cognitive-behavioural theories of grief (Boelen, van den Hout, & van den Bout, 2006a; Maccallum & Bryant, 2013; Shear & Shair, 2005), has shown that negative cognitions and avoidance behaviour may explain the debilitating impact of traumatic loss (Boelen et al., 2015; Mancini, Prati, & Black, 2011). According to Boelen et al.’s (2006a) cognitive-behavioural model, three processes play a crucial role in the onset and maintenance of post-loss psychopathology: insufficient integration of the loss into the autobiographical knowledge base, negative cognitions, and avoidance behaviour.

Insufficient integration is mainly an implicit process, but has an explicit counterpart, in the form of ‘a sense of unrealness’. Unrealness can be defined as a subjective sense of uncertainty or ambivalence about the irreversibility of the separation that is often expressed in phrases such as ‘I know that s/he is dead, but it feels as if it did not happen’ (Boelen, 2010). Unrealness may trigger intrusive memories of the loss and cause people to continue to feel shocked or stunned by the loss once they are confronted with reminders of the loss (Boelen, 2010; Boelen et al., 2006a). Negative cognitions about the future (e.g. ‘I don’t have confidence in the future’), life (e.g. ‘My life has no purpose anymore since he/she died’), the self (e.g. ‘Since he/she is not here anymore, I feel less worthy’), and catastrophic misinterpretations about one’s own reactions to the loss (‘If I would allow myself to really experience the grief, I will lose control’) have shown to be related to increased PG, PTS, and depression levels concurrently and longitudinally (Boelen, van den Bout, & van den Hout, 2006b; Boelen, van Denderen, & de Keijser, 2016). Avoidance behaviour is also associated with psychopathology following loss (Boelen & Eisma, 2015; Boelen & van den Bout, 2010), including depressive avoidance (i.e. withdrawal from previous fulfilling activities because of the belief that these activities are pointless since the loss) and anxious avoidance (i.e. avoidance of people, situations, or places that are related to the deceased out of the belief that confrontation with reminders of the loss is unbearable).

Cognitive-behavioural therapy (CBT), encompassing cognitive restructuring to change negative cognitions and exposure and behavioural activation to counter avoidance behaviours, is the treatment of choice for bereaved people (see for overviews Boelen & Smid, 2017a; Currier, Holland, & Neimeyer, 2010; Doering & Eisma, 2016). However, CBT yields small to moderate effect sizes (Currier et al., 2010) and only half of the bereaved people experience clinically relevant reductions in PG following CBT (Doering & Eisma, 2016). Accordingly, more research into the effectiveness of grief treatments is needed to adequately support bereaved persons at risk for psychopathology (Currier et al., 2010; Doering & Eisma, 2016). For instance, a limited number of controlled trials have evaluated the effectiveness of grief treatment (see for overviews Boelen, 2016; Boelen & Smid, 2017a; Rosner, 2015; Wittouck, van Autreve, De Jaegere, Portzky, & van Heeringen, 2011).

One such trial showed that CBT plus imaginal exposure led to greater reductions in PG and depression than CBT alone (Bryant et al., 2014). Confronting people with emotional memories of the loss using exposure seems critical in reducing post-loss psychopathology. A frequently applied intervention to reduce distress associated with a traumatic event is eye movement desensitization and reprocessing (EDMR). Meta-analyses have supported the effects of EMDR (Bisson et al., 2007; Bradley, Greene, Russ, Dutra, & Westen, 2005; Seidler & Wagner, 2006). In EMDR, people are asked to recall the most traumatic memories related to the traumatic event while they simultaneously make eye movements (in most cases by following the hand of the therapist). The effects of EMDR have been explained by the so-called ‘working memory theory’: by recalling a memory it becomes sensitive to change. By dual taxing the working memory (i.e. making eye movements and retrieving a memory), recourses for imagery of the memory are limited. Consequently, the memory will be less vivid and emotional during recalls in the future (van den Hout & Engelhard, 2011).

It has been proposed that EMDR might also be effective as a way of exposure to loss-related emotional memories, because it may facilitate integration of loss-related thoughts and memories with autobiographical knowledge (Solomon & Rando, 2012). EMDR might be particularly promising for people bereaved by a traumatic loss who are more susceptible to develop pervasive PTS levels than people confronted with a non-traumatic loss (Kaltman & Bonanno, 2003; Neria & Litz, 2004; Solomon & Rando, 2012). There is some evidence that eye movements during recall of loss-related memories reduce emotional reactivity in healthy bereaved students (Hornsveld et al., 2010). Further, a clinical trial showed larger treatment effects for EMDR compared to a behavioural-based treatment in terms of, among other things, reduction in PTS in people confronted with an unnatural and violent death (Sprang, 2001). In a randomized controlled trial (RCT) among homicidally bereaved people, cognitive therapy (CT) with EMDR resulted in clinically relevant reductions of PG and PTS levels compared with waiting list controls (van Denderen et al., in press).

We aim to conduct an RCT evaluating the effectiveness of CT with EMDR versus a waiting list control group in reducing PG, PTS, *and* depression levels in people exposed to a unique type of traumatic loss. People who lost one or multiple significant other(s) in the Ukrainian plane disaster will be studied. In this disaster, that took place on 17 July 2014, MH17 flight from Amsterdam to Kuala Lumpur crashed in Ukraine due to a missile impact. All 298 passengers were killed, including 193 Dutch citizens (Dutch Safety Board, 2015). Because this is a homogeneous sample of individuals, all confronted with losses due to the same manmade disaster, confounding effects of type and circumstances of the loss are ruled out. The CT part of the treatment aims to change maladaptive thoughts related to the (circumstances of the) losses; EMDR will be applied to reduce the vividness and distress of traumatic memories associated with the loss(es).

We expect that people in the treatment condition (i.e. CT with EMDR) will show larger reduction in PG, PTS, and depression levels compared to people waiting for treatment (i.e. ‘waiting list controls’; Hypothesis 1). Furthermore, we extend prior work by exploring possible mechanisms of change during treatment. We expect that greater reductions in PG, PTS, and depression levels will be related to greater reductions in a sense of unrealness, negative cognitions, avoidance behaviour, and intrusive memories (Hypothesis 2). Additionally, we expect that PG, PTS, and depression levels will decrease from pre-treatment to 12 weeks and 24 weeks post treatment (Hypothesis 3). Finally, at a micro-level, we anticipate that we will observe reductions in grief complaints during treatment based on measurements of these complaints in each treatment session (Hypothesis 4).

## 1. Method

### 1.1. Design

A multi-centre RCT will be conducted examining the effectiveness of CT and EMDR in reducing PG, PTS, and depression levels, in comparison with a waiting list control group. Participants in the intervention group will receive the treatment within one week after allocation. Participants of the waiting list control group after 12 weeks of waiting. Because this is one of the first RCTs examining the effects of EMDR for bereaved people, this design was chosen as optimal design given our resources. Furthermore, this design maximizes our recruitment efforts by ensuring that all participants receive treatment. Inclusion of the waiting list control group allows a treatment versus no treatment comparison which provides knowledge about the effects of CT and EMDR versus natural remission. Previous RCTs among bereaved people using a waiting list control condition showed no reasons for concerns about adverse effects (e.g. high dropout rate or significant increases in symptomology levels) of a waiting period (Eisma et al., 2015; Wagner, Knaevelsrud, & Maercker, 2006).

All participants will be instructed to fill in questionnaires pre-treatment and one week, as well as 12 weeks, and 24 weeks post-treatment. The participants of the waiting list control group will be asked to fill in one additional questionnaire during the last week of the waiting period, in order to examine the effect of the treatment versus no treatment. The study-protocol has been approved by the Medical Ethical Committee at the University Medical Center Groningen (UMCG) in the Netherlands (NL52722) and registered in the Dutch trial register (NTR5260).

### 1.2. Participants

Family members, spouses, colleagues, or friends of persons who died in the Ukrainian plane disaster are eligible to sign up for the study. Additional inclusion criteria are: (1) being 18 years or older, (2) being fluent in written and spoken Dutch, and (3) reporting clinical levels of PGD, PTSD, and/or depression as evidenced by scores above clinical cut-off points on relevant questionnaires (described below).

Most participants will be recruited during an on-going longitudinal survey study that started in May 2015 (Lenferink, de Keijser, Smid, Djelantik, & Boelen, 2017). In the first measurement occasion of this survey study, about one-year post-disaster, participants are asked whether they are interested in receiving information about a treatment study. If the participant responds positively to this question, a letter with information about the treatment study is sent along with the baseline-measure and an informed consent form. Additionally, we expect to recruit participants via media-attention (e.g. newspapers) and a website that has been developed with information about the treatment study (www.rouwnavliegrampmh17.nl). Recruitment of participants started in April 2016 and will continue until September 2017.

A potential participant will be excluded from participation in this study, if s/he is: (1) suffering from a substance use disorder, (2) suffering from a psychotic disorder, (3) mentally disabled (all based on clinical judgment at the intake), and/or (4) has a heightened risk of suicide (based on the highest score on item 12 (‘thoughts of dead or suicide’) of the Quick Inventory of Depressive Symptomatology – Self Report).

Potential participants will also be excluded from the study if they are receiving psychosocial professional support from a psychologist, psychiatrist, or other professional mental care worker at the time of entry into our study in order to rule out (combination-)effects of additional interventions. During the waiting period, participants in the waiting list control group are not allowed to receive other forms of psychosocial professional support, due to the same reason as described above. Based on a single item we will assess whether the participant received other forms of psychosocial professional support. However, for ethical reasons participants are allowed to (continue to) receive support from Victim Support the Netherlands (i.e. a non-governmental support organization that supports and monitors (i.e. watchful waiting) bereaved family members following the disaster). The psychosocial support received by participants after randomization will be registered.

People who wish to participate in the study and are not eligible due to subthreshold scores on the outcome measures or other reasons will be referred to Victim Support the Netherlands or their general practitioner and will be excluded from participation in the study.

### 1.3. Procedure

After receiving the filled-in baseline questionnaire and signed consent form potential participants will be screened for eligibility based on the inclusion and exclusion criteria. All participants will be randomly allocated to the intervention group or waiting list control group within one week after signing the consent form. An independent researcher carries out the stratified randomization procedure by using a random number generator (www.random.org). An allocation ratio of 1:1 will be applied. Stratification variables are gender, number of losses (i.e. single versus multiple), and the type of psychopathology that are present at clinical levels (PGD, PTSD, or depression versus comorbidity of these disorders). The results of the randomization will be communicated by e-mail or letter (depending on the way the questionnaire is delivered). Participants voluntarily withdrawing from the treatment will receive questionnaires at each measurement occasion. The costs that are related to the treatment (i.e. therapy costs and travel expenses) will be fully reimbursed by the Victim Fund.

### 1.4. Materials

Table 1 shows which measures were assessed at each time point.

**Table 1.** Overview of variables, concepts, measures, and time points.

Variable	Concept	Measure	Time point
Outcome	Prolonged grief levels	TGI-SR	T0, T0.1, T1, FU1, FU2
	Posttraumatic stress levels	PCL-5	T0, T0.1, T1, FU1, FU2
	Depression levels	QIDS-SR	T0, T0.1, T1, FU1, FU2
Possible mechanisms of change	A sense of unrealness	EUS	T0, T0.1, T1, FU1, FU2
	Severity of grief cognitions	GCQ	T0, T0.1, T1, FU1, FU2
	Severity of avoidance behaviour	DAAPGQ	T0, T0.1, T1, FU1, FU2
	Severity of intrusive memories	TMQ	T0, T0.1, T1, FU1, FU2
Other	Prolonged grief levels	B-TG	Start of each treatment session
	Sociodemographic information		T0

TGI-SR = Traumatic Grief Inventory Self Report; PCL-5 = Posttraumatic Stress Disorder Checklist for DSM-5; QIDS-SR = Quick Inventory of Depressive Symptomatology – Self Report; EUS = Experienced Unrealness Scale; GCQ = Grief Cognition Questionnaire; DAAPGQ = Depressive and Anxious Avoidance in Prolonged Grief Questionnaire; TMQ = Trauma Memory Questionnaire; B-TG = Brief Traumatic Grief questionnaire; T0 = baseline measure; T1 = post-treatment assessment; T0.1 = post-waiting period measure; FU1 = follow-up measure at 12 weeks; FU2 = follow-up measure at 24 weeks.

#### 1.4.1. Outcome measures

PG levels will be assessed with the Traumatic Grief Inventory Self Report version (TGI-SR; Boelen & Smid, 2017b). The TGI-SR measures symptoms of PGD (in accord with proposed ICD-11 criteria of Prigerson et al., 2009) and PCBD as included in the DSM-5 (American Psychiatric Association, 2013). The TGI-SR consists of 18 items (e.g. ‘I had trouble to accept the loss’) rated on 5-point scales with 1 = never and 5 = always. The instruction of the original questionnaire is altered from ‘the death of your loved one’ to ‘the death of your loved one(s) due to the Ukrainian plane disaster’. The TGI-SR has adequate psychometric properties (Boelen & Smid, 2017b). Participants are considered eligible for participation when they score 3 (3 = sometimes) or higher on at least one B-cluster symptom (item 1, 2, 3, and 14), and at least six C-cluster symptoms (items 4–11 and 15–18) and a score of 2 (2 = seldom) or higher on the D-cluster symptom (item 13), which is based on the DSM-5 diagnostic rule.

The severity of PTS levels will be assessed with the 20-item PTSD Checklist for DSM-5 (PCL-5; Blevins, Weathers, Davis, Witte, & Domino, 2015; Boeschoten, Bakker, Jongedijk, & Olff, 2014). Items (e.g. ‘In the past month, how much were you bothered by: Repeated, disturbing, and unwanted memories of the stressful experience?’) are rated on 5-point scales with 0 = not at all and 4 = extremely. In the instruction of the questionnaire we refer to ‘the death of your loved one(s) due to the Ukrainian plane disaster’ instead of ‘the stressful experience’. Psychometric properties of the PCL-5 are adequate (Blevins et al., 2015). Each item rated as 2 = moderately or higher will be considered as symptom endorsed. Clinical significant PTSD is defined in accord with the DSM-5 diagnostic rule (American Psychiatric Association, 2013), which requires one B item (items 1–5), one C item (items 6–7), two D items (items 8–14), and two E items (items 15–20).

Severity of depression complaints will be assessed with the 16-item Quick Inventory of Depressive Symptomatology (QIDS-SR; Rush et al., 2003). Participants are asked to choose one of four options (ranging from 0 to 3) indicating how frequently they experienced each symptom (e.g. ‘Feeling slowed down’) during the last week. The QIDS-SR has good psychometric properties (Rush et al., 2003). According to Rush et al. (2003), a score of ≥6 reflects mild depression levels and will therefore be used as inclusion criterion for participation in the current RCT.

#### 1.4.2. Possible mechanisms of change

The Experienced Unrealness scale is a 5-item measure of the sense of unrealness about the loss (Boelen, 2010). Participants rate their agreement with each item on 8-point scales with 0 = not at all true for me 7 = completely true for me. An example item is: ‘It feels unreal that [–] is gone forever’. Psychometric properties of this measure are adequate (Boelen, 2010).

The Grief Cognitions Questionnaire (GCQ) is a 38-item measure of loss-related negative thoughts developed by Boelen and Lensvelt-Mulders (2005). Four of its nine subscales will be used in our study (following the example of Boelen et al., 2015). Three subscales aim to measure global negative beliefs about the Self (six items, e.g. ‘Since [–] is dead, I feel less worthy’), Life (four items, ‘My life is meaningless since [–] died’), and the Future (five items, ‘I don’t have confidence in the future’), respectively. A fourth subscale represents Catastrophic Misinterpretations of one’s own grief reactions (four items, ‘Once I would start crying, I would lose control’). Participants rate their agreement with each item on 6-point scales with 0 = disagree strongly and 5 = agree strongly. Psychometric properties of this measure are adequate (Boelen & Lensvelt-Mulders, 2005).

The Depressive and Anxious Avoidance in Prolonged Grief Questionnaire (DAAPGQ) is a 9-item measure, with five items tapping depressive avoidance (e.g. ‘I avoid doing activities that used to bring me pleasure, because I feel unable to carry out these activities’) and four items tapping anxious avoidance (e.g. ‘I avoid situations and places that confront me with the fact that [–] is dead and will never return’). Participants rate their agreement with each item on a 8-point scale with 0 = not at all true for me and 7 = completely true for me. Psychometric properties of the subscales are adequate (Boelen & van den Bout, 2010).

The Trauma Memory Questionnaire (TMQ) consists of 13 items divided into two subscales (Halligan, Michael, Clark, & Ehlers, 2003). The 8-item subscale ‘Intrusion’ will be used in the current study. The items assess characteristics of traumatic memories (i.e. the extent to which trauma memories have strong perceptual elements and are accompanied by a sense of reliving the event). On 5-point scales ranging from 0 = not at all to 4 = very strongly, the participants rate their agreement with each item (e.g. ‘My memories of the event consist of vivid images’). We adapted the instruction of the questionnaire to refer to the loss of one or more significant others due to the plane disaster. The TMQ has adequate psychometric properties (Halligan et al., 2003).

#### 1.4.3. Other measures

The Brief Traumatic Grief questionnaire (B-TG) is a brief questionnaire with five items tapping PG developed for ongoing research on grief treatments in different samples. These data will be collected by the therapist during each session. On 5-point scales, with 1 = not at all and 5 = very strongly, the participants rate their agreement with each item (e.g. ‘I feel sadness’).

Socio-demographic characteristics of the participant (i.e. gender, age, educational level, country of birth) and the deceased relative(s) (i.e. gender, age, country of birth) and loss-related variables (number of lost relatives due to the plane disaster and kinship to the deceased) will be registered.

### 1.5. Treatment

The treatment consists of eight weekly sessions offered in a time period of maximum 12 weeks.^1^ In the first session, therapist and client introduce themselves, share expectations regarding the treatment, and the participant is invited to share the story about the deceased loved one(s). Social support is the central theme of the second session. The client is asked to invite a relative to join the client in this session. During sessions 3, 4, and 5, EMDR is offered. Sessions 6, 7, and 8 consist of changing maladaptive thoughts using conventional cognitive restructuring procedures. Each EMDR session has a duration of 90 minutes, all other sessions a duration of 60 minutes. Participants will receive a manual including psycho-education and exercises focused on identifying and altering maladaptive thoughts. Treatments are conducted by licensed therapists, divided over the Netherlands, who have received a one-day training about the treatment protocol. All therapists have ample experience in treating people confronted with traumatic loss (e.g. homicide, suicide, long-term disappearance) and will be supervised by the third, fourth, and fifth author.

### 1.6. Analyses

To test the first hypothesis, analyses of covariance (ANCOVA) will be conducted (Hox, Moerbeek, & van de Schoot, 2010; van Breukelen, 2006). PG, PTS, or depression levels post-treatment or post-waiting will be included as dependent variable, condition as independent variable, and baseline PG, PTS, or depression levels as covariate. To test hypotheses 2 and 3, the T0 data of the participants from the treatment condition will be combined with the T0.1 data of the waiting list control condition (i.e. ‘combined pre-treatment scores’) and condition will be included as covariate. Second, to test to what extent symptom improvement in treatment is related to improvement in possible mechanisms of change (Hypothesis 2), residual gain scores will be calculated for all outcome measures (i.e. PG, PTS, and depression) and possible mechanisms of change (i.e. a sense of unrealness, negative cognitions, avoidance behaviour, and intrusive memories), following previous work of van Minnen, Arntz, and Keijsers (2002) and Boelen, de Keijser, van den Hout, and van den Bout (2011). As recommended by Steketee and Chambless (1992), residual gain scores will be calculated by subtracting the standardized combined pre-treatment scores multiplied by the correlation between standardized combined pre-treatment scores and standardized post-treatment (or follow-up measures) scores from standardized post-treatment scores (or follow up measures). Multiple regression analyses will be conducted to examine the associations between residual gain scores of PG, PTS, or depression on the one hand and residual gain scores of a sense of unrealness, negative cognitions, avoidance behaviour, or intrusive memories on the other hand, while controlling for condition (i.e. immediate vs. delayed treatment). Third, long-term treatment effects (i.e. 12 and 24 weeks post-treatment) on the three outcome measures will be examined by including Time (dummy coded with the first measurement occasion as reference category) as main effect in multilevel models (Hypothesis 3). Finally, a multilevel analysis with Time (dummy coded with the first measurement occasion as reference category) as main effect will also be conducted to examine change in PG levels during treatment (using B-TG data; Hypothesis 4). In case multiple participants are nested in families of the same deceased person(s) a level will be added to the multilevel analyses. Effect sizes will be computed for change in PG, PTS, and depression levels at all time points. An intention-to-treat principle will be applied during the data analyses.

### 1.7. Sample size

Our sample size calculation is based on the main analysis to test Hypothesis 1. Based on previous grief trials (Currier et al., 2010 and van Denderen et al., in preparation), we expect to find a medium effect size difference between the conditions. With a power of 80% and α of 0.05, a sample size of at least 128 participants (64 per condition) is needed. By taking into account a dropout rate of 19% (based on the mean dropout rate as reported in Currier et al., 2010), a total sample size of at least 158 participants is required.

## 2. Discussion

The loss of one or more significant other(s) due to a manmade disaster is a unique type of traumatic loss. Being confronted with traumatic loss is associated with a higher risk for developing long-lasting psychological complaints, including PGD, PTS, and depression, compared with non-violent losses (e.g. illness; Boelen et al., 2015; Kaltman & Bonanno, 2003; Kloep et al., 2014). To the best of our knowledge, controlled clinical trials examining treatment effects or research into possible mechanisms of change in treatment among bereaved people are scarce. The present article describes the rationale of a RCT comparing CT with EMDR to a waiting list control group that aims to evaluate the effectiveness and potential mechanisms of change of treatment.

Strengths of this study are: (1) the inclusion of an intervention as well as a waiting list control group, (2) use of a homogeneous bereaved sample (e.g. same cause and circumstances of the loss), (3) the focus on three outcome measures (tapping symptoms of PGD, PTSD, and depression), (4) the preliminary evaluation of possible mechanisms of change of the treatment, and (5) process-monitoring of changes in PG levels on micro-level using session-by-session assessments. One of the limitations of this study is the reliance on self-report measures, which may overestimate symptom levels (Engelhard et al., 2007). In addition, we will assess baseline data at one time point, which may lead to overestimation of treatment effects due to regression toward the mean of participants with extreme scores (Biglan, Ary, & Wagenaar, 2000). Furthermore, the waiting list controls start with the treatment after 12 weeks of waiting. Therefore, we are only able to compare the treatment condition to the waiting list controls at the one week post-treatment assessment and not at the 12 weeks and 24 weeks follow-up assessments. Accordingly, the potential working mechanisms of change and the outcome variables will be measured concurrently, we will therefore be unable to examine mediation effects (Maxwell & Cole, 2007). Lastly, we will use an outreach method to recruit participants, which may limit the generalizability of future results to treatment-seeking bereaved people in general.

We expect that the results of this RCT will be valuable for clinical practice. Results are likely to enhance knowledge about the effectiveness of CT and EMDR for traumatically bereaved people, and the potential working mechanisms of such a treatment. The results of this study are also expected to be relevant for future research, which could further evaluate the potential effectiveness of CT and EMDR, for instance by comparing CT and EMDR with an active control condition, for instance grief-focused CBT (Boelen, de Keijser, van den Hout, van den Bout, 2007) or similar approaches (Peri, Hasson-Ohayon, Garber, Tuval-Mashiach, & Boelen, 2016; Rosner, 2015; Rosner, Bartl, Pfoh, Kotoučová, & Hagl, 2015; Smid et al., 2015) in different populations exposed to traumatic loss. To conclude, our results may contribute to refinement of evidence-based treatment options for bereaved people.

## Notes

1. Although previous RCTs evaluated at least 12 PG treatment sessions (see for an overview Wittouck et al., 2011), we chose to offer eight sessions, which accords with previous research indicating that eight sessions were sufficient to significantly reduce PG and PTS levels among homicidally bereaved people (van Denderen et al., in preparation).
